# Alkaloid Constituents of the Amaryllidaceae Plant *Amaryllis belladonna* L.

**DOI:** 10.3390/molecules22091437

**Published:** 2017-08-31

**Authors:** Luciana R. Tallini, Jean Paulo de Andrade, Marcel Kaiser, Francesc Viladomat, Jerald J. Nair, José Angelo S. Zuanazzi, Jaume Bastida

**Affiliations:** 1 Departament de Biologia, Sanitat i Medi Ambient, Facultat de Farmàcia, Universitat de Barcelona, Av. Joan XXIII 27–31, 08028 Barcelona, Spain; lucianatallini@gmail.com (L.R.T.); fviladomat@ub.edu (F.V.); nairjj@gmail.com (J.J.N.); 2 Departamento de Química Orgânica, Universidade Federal do Espírito Santo, Av. Fernando Ferrari 845, Victoria 29075-015, Brazil; deandradejp@gmail.com; 3 Medicinal Parasitology and Infection Biology, Swiss Tropical Institute, Socinstrasse 57, 4051 Basel, Switzerland; marcel.kaiser@unibas.ch; 4 University of Basel, Petersplatz 1, 4001 Basel, Switzerland; 5 Faculdade de Farmácia, Universidade Federal do Rio Grande do Sul, Av. Ipiranga 2752, Porto Alegre 90610-000, Brazil; zuanazzi@ufrgs.br

**Keywords:** *Amaryllis belladonna*, Amaryllidaceae, alkaloids, biosynthetic pathways, NMR, MS

## Abstract

The plant family Amaryllidaceae is well-known for its unique alkaloid constituents, which exhibit a wide range of biological activities. Its representative, *Amaryllis belladonna*, has a geographical distribution covering mainly southern Africa, where it has significant usage in the traditional medicine of the native people. In this study, *A. belladonna* samples collected in Brazil were examined for alkaloid content. Alkaloid profiles of *A. belladonna* bulbs were generated by a combination of chromatographic, spectroscopic and spectrometric methods, including GC–MS and 2D NMR. In vitro screening against four different parasitic protozoa (*Trypanosoma cruzi*, *T. brucei rhodesiense*, *Leishmania donovani* and *Plasmodium falciparum*) was carried out using the *A. belladonna* crude methanol extract, as well as three of its alkaloid isolates. Twenty-six different Amaryllidaceae alkaloids were identified in the *A. belladonna* bulb samples, and three of them were isolated. Evidence for their respective biosynthetic pathways was afforded via their mass-spectral fragmentation data. Improved data for 1-*O*-acetylcaranine was provided by 2D NMR experiments, together with new ^1^H-NMR data for buphanamine. The crude extract and 3-*O*-acetylhamayne exhibited good antiprotozoal activity in vitro, although both with a high cytotoxic index.

## 1. Introduction

The plant family Amaryllidaceae has attracted considerable attention in view of the fascinating structural features and varied biological activities manifested by its alkaloid principles. Anticancer, antimicrobial and anticholinesterase activities represent some of these biological properties [1]. The genus *Amaryllis* L. comprises two species, *A. belladonna* and *A. acuminata*, both native to southern Africa [2]. Also referred to by the folk names “belladonna-lily” and “naked-lady”, *A. belladonna* has been used for several centuries in the medicinal traditions of the Sotho, Xhosa and Zulu peoples of South Africa, and in Java for the treatment of “swelling” (a presumed synonym for cancer) [2,3].

The characteristic isoquinoline alkaloids produced by members of the Amaryllidaceae are derived from the aromatic amino acids phenylalanine and tyrosine, which combine to give the common precursor *O*-methylnorbelladine [4]. Consequently, alternative ways of oxidative phenolic coupling of this key intermediate metabolite lead to the three main discernible skeleton types that form the basis for further structural diversity in the Amaryllidaceae. The *ortho–para*´ coupling of *O*-methylnorbelladine results in the formation of the lycorine-type skeleton, which in turn leads to homolycorine-type compounds. The galanthamine-type skeleton originates from *para–ortho*´ phenolic oxidative coupling, whilst *para–para*´ coupling gives the crinine-, haemanthamine-, tazettine-, narciclasine- and montanine-type structures [5].

Gas chromatography-mass spectrometry (GC–MS) studies of Amaryllidaceae plants show that they contain a quite variable number of alkaloids [6,7]. Each species produces a mixture of alkaloids, often with a few dominant compounds and a larger number of compounds at lower concentrations, which is likely to result from differences in substrate specificity and expression level of the various biosynthetic enzymes [8]. The evolution of the biosynthetic pathways in the production of Amaryllidaceae alkaloids remains unclear [9]. Most research on Amaryllidaceae alkaloid biosynthesis was carried out during the 1960s and early 1970s. More recently, there has been notable interest in the biosynthesis of galanthamine and its congeners, given their success as commercial drug targets [10]. In this regard, in 2001, galanthamine was approved by the FDA (Food and Drug Administration) for the clinical management of mild to moderate Alzheimer’s disease [11]. Subsequently, there has been widespread interest in the Amaryllidaceae as a source of novel chemotherapeutics [12].

Thirty-one alkaloids have been previously identified in *A. belladonna*, including 1-*O*-acetylcaranine, 6-hydroxybuphanisine, 6-hydroxycrinine, 8-demethylmaritidine, 11-hydroxyvittatine, amarbellisine, amaryllidine, amaryllisine, ambelline, anhydrolycorinone, anhydrolycorinium, belladine, bellamarine, buphanamine, caranine, crinine, galanthamine, galanthine, hippadine, hippeastrine, ismine, lycorenine, lycorine, pancracine, parkacine, parkamine, powelline, pratorimine, pratosine, undulatine and vittatine [2,13,14,15,16,17,18,19,20,21]. Anti-bacterial, anti-fungal and anti-neoplastic effects are associated with some of the alkaloids isolated from *A. belladonna* [2,20].

Protozoal diseases such as leishmaniasis, trypanosomiasis and malaria are still the most prevalent parasitic diseases worldwide, with a significant social and economic impact [22]. In many cases, contemporary chemotherapeutic regimens are not satisfactory due to a lack of effectiveness as well as the toxicity associated with long-term treatment. Furthermore, the emergence of global drug resistance has necessitated the development of new anti-parasitological drugs [23,24].

The aim of this work was to perform a detailed study of the alkaloid constituents of *A. belladonna* utilizing spectroscopic and chromatographic methods, including GC–MS and NMR. In this way, twenty-six alkaloids were identified and evidence presented for their respective biosynthetic pathways. Furthermore, a comprehensive NMR study of 1-*O*-acetylcaranine is presented, together with new ^1^H-NMR data of buphanamine. Finally, anti-parasitological activities of the crude extract and three isolated alkaloids from *A. belladonna* are reported for the first time.

## 2. Results and Discussion

### 2.1. Alkaloids Identified in A. belladonna by GC–MS

The structural types of the alkaloids from *A. belladonna* were identified by comparing their GC–MS spectra and Kovats retention index (RI) values with those of authentic Amaryllidaceae alkaloids previously isolated and identified by spectrometric methods as nuclear magnetic resonance (NMR), ultraviolet (UV), circular dichroism (CD) and mass spectrometry (MS) in our laboratory. The NIST 05 Database and literature data were also used (Table 1). The MS spectra were deconvoluted by AMDIS 2.64 software (NIST).

Fourteen of the identified alkaloids were detected for the first time in this species: trisphaeridine [2], buphanisine [3], anhydrolycorine [4], 11,12-dehydroanhydrolycorine [10], 6-methoxybuphanidrine [11], 3-*O*-acetylvittatine [12], buphanidrine [14], 1-*O*-acetyllycorine [17], 3-*O*-acetylpowelline [18], 6-hydroxybuphanidrine [21], 11-*O*-acetylambelline [22], 3-*O*-acetylhamayne [24], crinamidine [25] and distichamine [26]. All the alkaloids found in *A. belladonna* belong to the typical Amaryllidaceae alkaloid groups, including the lycorine, crinine and haemanthamine groups. 1-*O*-Acetylcaranine [6] was the most abundant alkaloid and the crinine group was the most represented, whilst no galanthamine-type alkaloids were detected in this plant.

**Figure 1:**
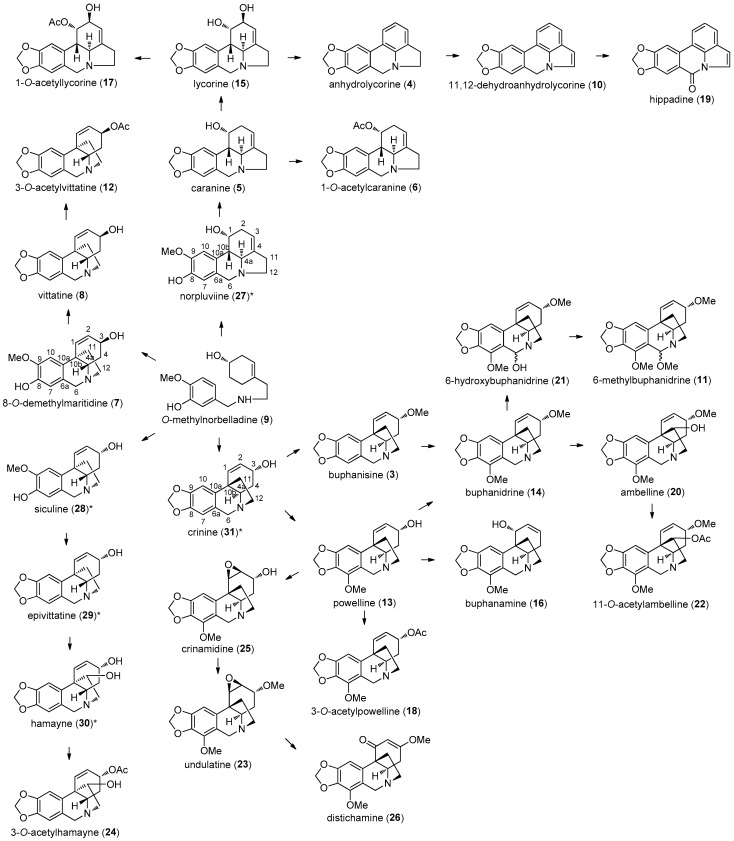
Tentative biosynthetic routes to the alkaloids of *A. belladonna*. * Alkaloids not identified in this plant but which could be involved in the metabolic route include norpluviine (**27**), siculine (**28**), epivittatine (**29**), hamayne (**30**) and crinine (**31**).

Based on their structural identities, it has been possible to show how the alkaloids identified in *A. belladonna* could be linked biosynthetically (Figure 1). We propose that the chemical similarity of the alkaloid types from this species may be due to different combinations of common biosynthetic reactions, such as hydroxylation, hydrogenation, reduction, epoxidation, methylation, methoxylation, acetylation and allylic rearrangements, starting from a common precursor. Precedence for these reactions has been studied in other alkaloid groups; for example, indole alkaloids in *Catharanthus roseus* (Apocynaceae) [26,27].

To date, galanthamine metabolization represents the most studied Amaryllidaceae alkaloid biosynthetic pathway. Experiments involving the application of ^13^C-labelled *O*-methylnorbelladine to field-grown *Leucojum aestivum* plants indicate that galanthamine biosynthesis involves phenolic oxidative coupling of *O*-methylnorbelladine to a dienone, the adduct of which undergoes spontaneous conversion to *N*-demethylnarwedine. The latter is converted via stereoselective reduction to *N*-demethylgalanthamine, which is then *N*-methylated to galanthamine [10].

Benzylamine *O*-methylnorbelladine (**9**) was also shown to be a precursor of norpluviine (**27**), which is known to be integral to lycorine-type alkaloid metabolization [28]. Apart from this, the transformation of caranine (**5**) into lycorine (**15**) has been observed in both *Zephyranthes candida* and *Clivia miniata* [29,30]. In *A. belladonna*, it is conceivable that 1-*O*-acetylcaranine (**6**) and 1-*O*-acetyllycorine (**17**) are derived via acetylation of the C-1 hydroxy group in caranine (**5**) and lycorine (**15**), respectively. Furthermore, we suggest that lycorine (**15**) could be metabolized to anhydrolycorine (**4**) by double dehydration of the C-1 and C-2. Also, we suggest that anhydrolycorine (**4**) may be a precursor of 11,12-dehydroanhydrolycorine (**10**) by reduction of the D-ring double bond, which could then serve as a precursor to hippadine (**19**) via hydroxylation at C-6 and subsequent oxidation to the amide functionality.

Crinine (**31**) can be considered the precursor of all crinine-type alkaloids in possession of the methylenedioxy group, whilst buphanisine (**3**) and powelline (**13**) may be easily derived from crinine (**31**) by methylation and methoxylation reactions at C-3 and C-7, respectively. In addition, C-7 methoxylation of buphanisine (**3**) and/or C-3 methylation of powelline (**13**) are possible alternative steps in the buphanidrine (**14**) biosynthetic pathway. The presence of ambelline (**20**) and 11-acetylambelline (**22**) suggests a C-11 hydroxylation of buphanidrine (**14**) followed by an acetylation reaction. Buphanidrine (**14**) is a likely precursor of 6-hydroxybuphanidrine (**21**), which could then lead to 6-methoxybuphanidrine (**11**) via a methylation reaction. Also in the crinine group, it is conceivable that metabolic acetylation of powelline (**13**) would afford 3-*O*-acetylpowelline (**18**). Powelline (**13**) could also facilitate crinamidine (**25**) metabolization via C-2, C-3 epoxidation. This would subsequently lead to undulatine (**23**) by C-3 methylation, which in turn is distinguished as the precursor to distichamine (**26**) following allylic rearrangement. Further to this, 8-*O*-demethylmaritidine (**7**) is a plausible precursor of vittatine (**8**) via methylenedioxy group transformation, from which 3-*O*-acetylvittatine (**12**) may be readily accessible. The presence of 3-*O*-acetylhamayne (**24**) suggests that the structurally related alkaloids siculine (**28**), epivittatine (**29**) and hamayne (**30**) may also be present, but in hitherto undetectable quantities in *A. belladonna*.

### 2.2. 1-O-Acetylcaranine

**Figure 2:**
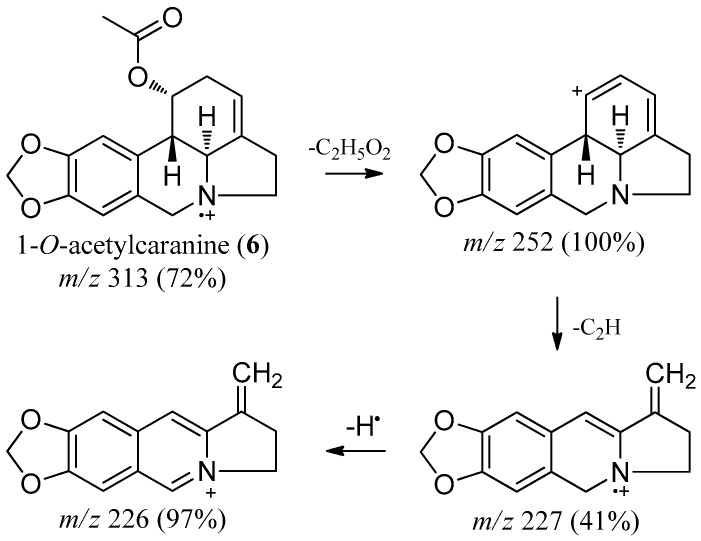
Mass fragmentation pattern of 1-*O*-acetylcaranine (**6**).

The base ion signal that occurs at *m*/*z* 252 in 1-*O*-acetylcaranine (**6**) is characteristic of deacetylation at C-1 and dehydration at C-1 and C-2 (Figure 2). Herein we present the complete NMR data for 1-*O*-acetylcaranine (**6**) by both 1D and 2D NMR spectroscopic analysis (see 1-*O*-acetylcaranine NMR spectra in Supplementary Materials). The ^1^H-NMR spectrum (Table 2) was similar to that of 1-*O*-acetylcaranine and caranine [13,31,32]. The shift of the H-1 proton signal to a lower magnetic field than that observed for caranine suggested a substitution of the hydroxyl group at C-1, which was further substantiated by the presence of a singlet at δ 1.93, indicative of an acetyl group. The ^13^C-NMR spectrum showed a singlet resonance signal at δ 171.0, which confirmed the presence of one carbonyl group. The COSY spectrum showed an allylic coupling between H-3 and H-4a and between H-3 and H-11, which allowed us to determine the H-10b proton location in the ^1^H-NMR spectrum. In addition, the small magnitude of the coupling constants between H-1 and H-10b allowed us to assign the α-orientation to the acetyl group. The two C-6 protons were differentiated as an AB system with a geminal coupling of around 14 Hz. H-4a showed NOESY correlations with both H-2α and H-6α, which turned out to be key correlations in the assignment of their orientation. Furthermore, H-12α was ascribable to a higher field as a consequence of NOESY contour correlation with H-6α. The HMBC spectrum allowed us to assign the quaternary carbons C-6a (δ 129.5) and C-10a (δ 127.8) via three-bond correlation with H-10 and H-7, respectively. The aromatic protons were ascribable to H-7 and H-10 due to three-bond HMBC correlations with C-10a and C-6a, respectively, in addition to NOESY correlations observed for the H-6/H-7 and H-1/H-10 proton pairs. The C-8 quaternary carbon (δ 146.2) was located via four-bond HMBC connectivity with both H-6, as well as via three-bond connectivity with H-10. The quaternary carbon C-9 (δ 146.5) was determined via three-bond HMBC connectivity with H-7. Finally, the singlet resonance signal at δ 139.26 was assigned to C-4, taking into account three bond connectivities to H-12β. All these data are in agreement with the structure of 1-*O*-acetylcaranine (**6**) (see Figure 4).

### 2.3. Buphanamine

**Figure 3:**
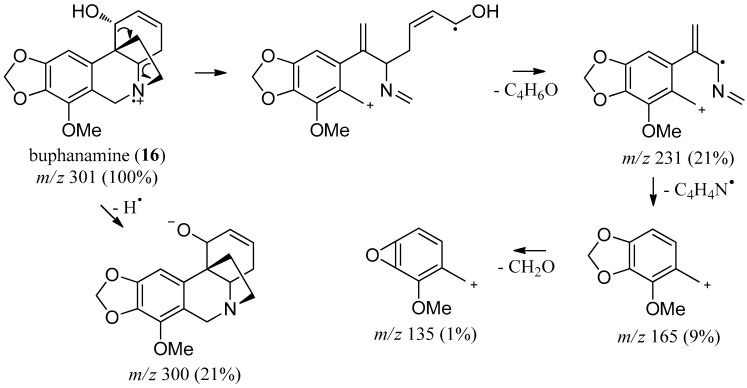
Mass fragmentation pattern for buphanamine (**16**) (adapted from [33]).

For most crinine-type alkaloids, the molecular ion is prominent as the base peak, and the fragmentation mechanism is initiated by opening of the C-11/C-12 ethano-bridge, indicating bond cleavage at the position β to the nitrogen atom (Figure 3) [33].

The ^1^H-NMR data we obtained for buphanamine (**16**) (Table 3) differed slightly from the data available in the literature [34]. The discrepancies we noted relate to the H-4α, H-4β, H-4a, H-6α, H-6β, H-11endo, H-11exo, H-12endo and H-12exo proton resonances (Δ +0.26, +0.22, +0.36, +0.26, +0.27, +0.14, +0.20, +0.24, +0.51, respectively). These are quite downfield-shifted compared to the corresponding data we obtained in our NMR analysis (see buphanamine NMR spectra in Supplementary Materials). Quaternization of the nitrogen atom via *N*-oxide or salt formation is known to influence the chemical shifts of protons in its vicinity [1].

**Figure 4:**
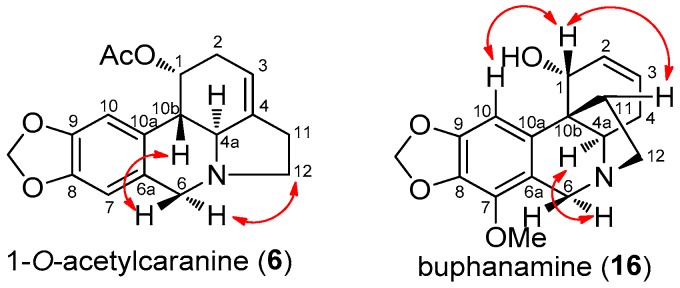
Key NOESY correlations observed for 1-*O*-acetylcaranine (**6**) and buphanamine (**16**).

In the ^1^H-NMR spectrum, the coupling constants between H-1 and H-2 (*J* = 5.5 Hz), H-2 and H-3 (*J* = 10.0 Hz), H-3 and H-4α (*J* = 4.5 Hz), together with the geminal coupling of around 19.7 Hz between H-4α and H-4β, allowed us to place the hydroxyl group at C-1 and the double bond between C-2 and C-3. The NOE contour between H-1 and 2H-11, and the homoallylic coupling between H-1 and H-4β, confirmed the α-orientation for the hydroxyl group. Interestingly, the COSY correlation observed between H-2 and 2H-4 also confirmed the allylic coupling between these protons. The two H-6 protons were clearly differentiated as part of an AB system, each with a geminal coupling value of 17.2 Hz. The singlet aromatic proton resonance was ascribable to H-10 due to three-bond HMBC correlations with C-6a and C-10b, in addition to a NOESY correlation with H-1, allowing the aromatic methoxyl group to be placed at C-7. H-6α was assigned to a lower field via a NOESY contour correlation with H-4a (Figure 4). The H-11exo and H-12exo protons were assignable based on the large values of their respective coupling constants. The quaternary carbons C-6a and C-10a were ascribed by means of their three-bond HMBC correlations with H-10 and H-1, respectively. Finally, the singlet resonance signal at δ = 48.3 was assigned to C-10b, taking into account three-bond connectivities to H-10, H-4α and H-4β. The absolute configuration of this alkaloid was determined from the CD spectrum, wherein the curve was qualitatively similar to that of buphanamine (**16**) [35].

### 2.4. Biological Activity

The biological activity tests against the parasitic protozoa and for cytotoxicity were performed as described earlier [36]. 3-*O*-Acetylhamayne showed higher activity than the other alkaloids against all protozoan parasites tested. It was active against *Trypanosoma brucei rhodesiense* (IC_50_ = 1.51 µg mL^−1^), *T. cruzi* (IC_50_ = 8.25 µg mL^−1^), *Leishmania donovani* (IC_50_ = 17.91 µg mL^−1^) and *Plasmodium falciparum* (IC_50_ = 1.14 µg mL^−1^). However, the cytotoxicity against L6 cells (rat skeletal myoblasts, IC_50_ = 1.72 µg mL^−1^) highlights that 3-*O*-acetylhamayne is not a selective antiparasitic agent. The crude extract exhibited activity against *T. brucei rhodesiense* (IC_50_ = 4.67 µg mL^−1^), *T. cruzi* (IC_50_ = 34.86 µg mL^−1^) and *P. falciparum* (IC_50_ = 1.17 µg mL^−1^), but it was less cytotoxic than 3-*O*-acetylhamayne. There is very little information about the structure–antiprotozoal activity relationship of the Amaryllidaceae alkaloids, but some results suggest that the methylenedioxy group can contribute to increase the antiprotozoal activity in these alkaloids [24]. The crude extract results suggest that *A. belladonna* could be an interesting source of alkaloids with antiparasitic activity. The antiprotozoal activity of *A. belladonna* alkaloids is summarized in Table 4.

## 3. Materials and Methods

### 3.1. Plant Material

Bulbs of *Amaryllis belladonna* L. were collected in Canela (Rio Grande do Sul, Brazil) in August 2014. The first sample was authenticated by Dr. Julie H. A. Dutilh, University of Campinas (Unicamp, Campinas, Brazil). A specimen voucher (179860) has been deposited in the Herbarium of the Universidade Federal do Rio Grande do Sul (UFRGS, Porto Alegre, Brazil).

### 3.2. Equipment

About 2 mg of each alkaloid extract was dissolved in 1 mL of MeOH and/or CHCl_3_ and injected directly into the GC–MS apparatus (Agilent Technologies 6890N coupled with MSD5975 inert XL) operating in the electron ionization (EI) mode at 70 eV. A Sapiens-X5 MS column (30 m × 0.25 mm i.d., film thickness 0.25 µm) was used. The temperature gradient performed was as follows: 12 min at 100 °C, 100–180 °C at 15 °C/min, 180–300 °C at 5 °C/min and 10 min hold at 300 °C. The injector and detector temperatures were 250 and 280 °C, respectively, and the flow-rate of carrier gas (He) was 1 mL/min. A split ratio of 1:5 was applied and the injection volume was 1 µL.

^1^H- and ^13^C-NMR, COSY, NOESY, HMQC and HMBC spectra were recorded on a Varian VNMRS 500 MHz using CDCl_3_ as the solvent and TMS as the internal standard. Chemical shifts were reported in units of δ (ppm) and coupling constants (*J*) expressed in Hz. CD, UV and IR spectra were recorded on Jasco-J-810, Dinko UV2310 and Thermo Scientific Nicolet iN10 MX spectrophotometers, respectively. High-resolution electrospray ionization mass spectrometry (HR–ESI–MS) spectra were obtained on an LC/MSD-TOF (2006) mass spectrometer (Agilent Technologies). Silica gel SDS chromagel 60 A CC (6–35 µm) was used for vacuum liquid chromatography (VLC), and silica gel 60 F254 Macherey-Nagel for analytics and prep. Spots on chromatograms were detected under UV light (254 nm) and by Dragendorff’s reagent stain.

### 3.3. Extraction

Fresh bulbs (2.5 kg) of *A. belladonna* were dried at 40 °C for seven days. After this, the dried material (950 g) was macerated with MeOH (4 × 2 L) at room temperature for 48 h, the combined macerate was filtered and the solvent evaporated to dryness under reduced pressure. The bulb crude extract (105 g) was then acidified to pH 3 with diluted H_2_SO_4_ (5% *v*/*v*) and the neutral material removed with Et_2_O (10 × 200 mL). The aqueous solution was basified up to pH 9–10 with NH_4_OH (25% *v*/*v*) and extracted with *n*-Hexane (12 × 200 mL) to give the *n*-hexane extract (0.47 g), which was followed by extraction with EtOAc (15 × 200 mL) to provide the EtOAc extract (4.07 g).

The extracts were subjected to a combination of chromatographic techniques, including vacuum liquid chromatography (VLC) [25] and semi-preparative thin-layer chromatography (TLC). The general VLC procedure consisted of the use of a silica gel 60 A (6–35 µm) column with a height of 4 cm and a variable diameter according to the amount of sample (2.5 cm for 400–1000 mg; 1.5 cm for 150–400 mg). Alkaloids were eluted with *n*-hexane containing increasing EtOAc concentrations, followed by neat EtOAc, which was gradually enriched with MeOH (reaching a maximum concentration of 20% *v*/*v*). Fractions of 10–15 mL were collected, monitored by TLC (UV 254 nm, Dragendorff’s reagent) and combined according to their profiles. For semi-preparative TLC, silica gel 60F_254_ was used (20 cm × 20 cm × 0.25 mm) together with different solvent mixtures depending on each particular sample (EtOAc:MeOH, 9:1 *v*/*v*, or EtOAc:MeOH, 8:2 *v*/*v*), always in an environment saturated with ammonia. The alkaloids were each identified by GC–MS and the three known alkaloids were isolated and structurally elucidated by NMR as 1-*O*-acetylcaranine (362.9 mg), 3-*O*-acetylhamayne (2.2 mg) and buphanamine (17.2 mg).

### 3.4. Characterization of Compounds

*1-O-Acetylcaranine* (**6**). ${\left[\alpha \right]}_{D}^{22}$ −161.84 (*c* 0.0009, CHCl_3_); UV (MeOH) λ_max_ (log ε): 290.5 (3.77), 235.5 (3.76) nm; IR (CHCl_3_) ν_max_ 2888, 1726, 1505, 1487, 1487, 1450, 1376, 1238, 1033, 934, 847 cm^−1^; ^1^H-NMR (CDCl_3_, 500 MHz) and ^13^C-NMR (CDCl_3_, 125 MHz) see Table 2; ESI–MS data shown in Table 1; HR–ESI–MS of [M + H]^+^ *m*/*z* 314.1394 (calcd. for C_18_H_20_NO_4_, 314.1387).

*Buphanamine* (**16**). ${\left[\alpha \right]}_{D}^{22}$ −169.03 (*c* 0.0009, CHCl_3_); UV (MeOH) λ_max_ (log ε): 285.0 (3.17), 207.0 (4.14) nm; CD (MeOH, 20 °C) Δε_250_ +1792, Δε_268_ −247, Δε_288_ +293; IR ν_max_ 3334, 2923, 1619, 1478, 1240, 1080, 1044, 753 cm^−1^; ^1^H-NMR (CDCl_3_, 500 MHz) and ^13^C-NMR, 125 MHz) see Table 3; ESI–MS data shown in Table 1; HR–ESI–MS of [M + H]^+^ *m*/*z* 302.1385 (calcd. for C_17_H_20_NO_4_, 302.1387).

## Figures and Tables

**Table 1:** Alkaloids identified in *A. belladonna* by GC–MS.

Alkaloid	RI	M^+^	MS
ismine (**1**)	2288.1	257 (28)	239 (20), 238 (100), 225 (7), 211 (7), 196 (9), 180 (8), 139 (8)
trisphaeridine (**2**)	2305.0	223 (100)	224 (15), 222 (38), 193 (2), 164 (15), 138 (26), 111 (14)
buphanisine (**3**)	2447.5	285 (100)	270 (34), 254 (34), 242 (12), 227 (24), 215 (85), 201 (23), 185 (22), 172 (22), 157 (33), 128 (33)
anhydrolycorine (**4**)	2470.2	251 (42)	250 (100), 220 (3), 192 (18), 163 (3), 125 (2)
caranine (**5**)	2560.9	271 (59)	270 (37), 252 (53), 240 (10), 227 (44), 226 (100), 212 (6), 181 (1)
1-*O*-acetylcaranine (**6**)	2569.6	313 (72)	270 (3), 252 (100), 240 (9), 227 (41), 226 (97)181 (2)
8-*O*-demethylmaritidine (**7**)	2575.5	273 (100)	256 (13), 244 (12), 230 (26), 216 (14), 201 (90), 189 (61), 175 (28), , 157 (19), 141 (11), 128 (24)
vittatine (**8**)	2578.0	271 (100)	254 (11), 242 (11), 228 (22), 214 (13), 199 (58), 187 (63), 173 (24), 157 (18), 141(13), 128 (29)
*O*-methylnorbelladine (**9**)	2578.5	273 (-)	166 (36), 137 (100), 122 (8), 94 (6)
11,12-dehydroanhydrolycorine (**10**)	2638.1	249 (59)	248 (100), 218 (1),190 (26), 163 (8), 137 (1), 123 (5), 95 (13)
6-methoxylbuphanidrine (**11**)	2656.8	345 (58)	330 (33), 298 (14), 287 (32), 259 (68), 227 (30), 181 (10), 145 (13)
3-*O*-acetylvittatine (**12**)	2690.6	313 (100)	298 (1), 284 (6), 270 (27), 254 (75), 252 (61), 224 (38), 216 (60), 198 (36), 187 (36), 139 (15), 128 (33)
powelline (**13**)	2702.7	301 (100)	284 (10), 272 (14), 258 (39), 244 (24), 229 (8), 217 (7), 202 (22), 187 (23), 159 (13), 143 (11), 127 (19)
buphanidrine (**14**)	2705.2	315 (100)	300 (31), 284 (35), 272 (9), 260 (39), 245 (69), 228 (24), 215 (21), 202 (20), 187 (18), 130 (18)
lycorine (**15**)	2722.0	287 (18)	286 (10), 268 (19), 250 (16), 238 (7), 227 (78), 226 (100), 211 (6), 147 (5), 119 (3)
buphanamine (**16**)	2726.5	301 (100)	282 (21), 272 (13), 256 (22), 244 (11), 190 (7), 165 (9)
1-*O*-acetyllycorine (**17**)	2754.9	329 (31)	268 (31), 250 (20), 227 (64), 226 (100), 211 (6), 192 (3), 167 (3), 147 (6)
3-*O*-acetylpowelline (**18**)	2768.7	343 (100)	314 (6), 300 (25), 284 (78), 283 (24), 268 (9), 254 (21), 246 (81), 228 (39), 217 (18), 202 (6), 183 (9)
hippadine (**19**)	2775.0	263 (100)	233 (2), 205 (7), 177 (24), 150 (13), 131 (9), 111 (2), 75 (7)
ambelline (**20**)	2814.6	331 (86)	316 (8), 299 (44), 287 (100), 270 (33), 260 (76), 257 (58), 239 (52), 228 (20), 211 (62), 190 (39)
6-hydroxybuphanidrine (**21**)	2828.4	331 (44)	314 (5), 300 (10), 282 (7), 276 (100), 261 (33), 258 (13), 243 (12), 228 (8), 217 (21), 216 (22)
11-*O*-acetylambelline (**22**)	2850.2	373 (100)	314 (58), 313 (50), 282 (46), 258 (48), 255 (36), 254 (63), 241 (38), 240 (35), 218 (44)
undulatine (**23**)	2855.2	331 (100)	316 (9), 300 (9), 286 (15), 258 (33), 244 (14), 232 (17), 217 (28), 205 (60), 189 (32), 173 (30)
3-*O*-acetylhamayne (**24**)	2907.7	329 (3)	300 (100), 269 (6), 240 (16), 224 (8), 212 (34), 211 (14), 199 (6), 181 (54), 167 (3), 153 (18), 128 (10)
crinamidine (**25**)	2954.3	317 (59)	300 (2), 288 (100), 258 (21), 244 (27), 230 (16), 217 (35), 203 (34), 189 (20), 173 (40), 145 (17)
distichamine (**26**)	2984.3	329 (100)	328 (25), 314 (15), 300 (9), 286 (9), 269 (5), 256 (3), 231 (20), 204 (13), 190 (5), 130 (2)

**Table 2:** ^13^C-NMR, ^1^H-NMR and HMBC data for 1-*O*-acetylcaranine (**6**) (500 MHz, CDCl_3_).

Position	δ_C_, Type	δ_H_ (*J* in Hz)	HMBC
1	66.60 d	5.84 br dd (4.5, 2.0)	C-3, C-4a, C-10b, OCOCH_3_
2α	33.55 t	2.39 m	
2β	33.55 t	2.64 m	
3	114.43 d	5.39 dd (4.9, 2.4)	C-4a, C-1
4	139.56 s		
4a	61.51 d	2.82 d (10.8)	
6α	57.09 t	3.54 d (14.3)	C-10a
6β	57.09 t	4.13 d (14.1)	C-4a, C-7, C-10a
6a	129.54 s		
7	107.34 d	6.57 br s	C-6, C-9, C10a
8	146.24 s		
9	146.49 s		
10	105.25 d	6.72 d (1.0)	C-6a, C-8, C-10b
10a	127.79 s		
10b	43.56 d	2.67 m	
11α	28.70 t	2.61 m	
11β	28.70 t	2.61 m	
12α	53.83 t	2.39 m	C-6
12β	53.83 t	3.33 ddd (9.4, 4.9, 4.4)	C-4, C-4a, C-6
OCH_2_O	101.16 t	5.91 d (1.5)–5.92 d (1.5)	
OCOCH_3_	171.03 q	1.93 s	
OCOCH_3_	21.53 q	1.93 s	

**Table 3:** ^13^C-NMR, ^1^H-NMR and HMBC data of buphanamine (**16**) (500 MHz, CDCl_3_).

Position	δ_C_, Type	δ_H_ (*J* in Hz)	HMBC
1	64.5 d	4.74 d (5.5)	C-3, C-4a, C-10a, C-11
2	125.5 d	6.00 dddd (10.0, 5.5, 2.8, 1.8)	C-10b
3	128.9 d	5.86 ddd (10.0, 4.5, 2.8)	C-4a
4α	28.2 t	2.57 dddd (19.7, 8.1, 4.5, 1.9)	C-2, C-10b
4β	28.2 t	1.99 dddd (19.8, 8.6, 1.1, 0.3)	C-2, C-10b
4a	59.2 d	3.43 t (8.3)	C-10a
6a	117.9 s		
6α	56.9 t	4.17 d (17.2)	C-4a, C-7, C-10a
6β	56.9 t	3.81 d (17.2)	C-4a, C-7, C-10a
7	140.8 s		
8	133.6 s		
9	148.6 s		
10	98.0 d	6.59 s	C-6a, C-8, C-10b
10a	137.0 s		
10b	48.3 s		
11endo	38.7 t	1.85 dddd (11.9, 8.8, 3.1, 1.3)	C-4a, C-10a
11exo	38.7 t	1.92 ddd (12.1, 10.0, 7.3)	C-4a, C-10a
12endo	51.4 t	2.75 ddd (13.0, 8.8, 7.2)	C-4a, C-6
12exo	51.4 t	3.40 ddd (13.0, 10.0, 3.0)	C-6, C-10b
OCH_2_O	100.7 t	5.88 d (1.5)–5.89 d (1.5)	C-8, C-9
OCH_3_	59.1 q	3.98 s	C-7

**Table 4:** In vitro antiprotozoal and cytotoxic activities of *A. belladonna*.

Parasite	*T. b. rhodesiense*	*T. cruzi*	*L. donovani*	*P. falciparum*	Cytotoxicity
Stage	Trypamastigotes	Amastigotes	Amastigotes	IEF (Intraerythrocytic)	
Strain	STIB 900 (IC_50_^a^)	Tulahuen C4 LacZ (IC_50_^a^)	MHOM-ET-67/L82 (IC_50_^a^)	NF54 (IC_50_^a^)	L6 (IC_50_^a^)
melarsoprol	0.0035 ^b^ and 0.0010 ^c^				
benznidazole		0.660 ^b^ and 1.080 ^c^			
miltefosine			0.085 ^b^ and 0.091 ^c^		
chloroquine				0.002 ^b,c^	
podophyllotoxin					0.005 ^b^ and 0.007 ^c^
1-*O*-acetylcaranine (**6**)	1.97	35.9	>100	3.21	14.2
3-*O*-acetylhamayne (**24**)	1.51	8.3	17.9	1.14	1.72
buphanamine (**16**)	28.2	62.9	>100	25.9	>100
crude extract	4.67	34.9	>100	1.17	34.3

^a^ all values expressed as μg mL^−1^; ^b^ reference value to 1-*O*-acetylcaranine; ^c^ reference value to buphanamine, 3-*O*-acetylhamayne and crude extract.

## Supplementary Materials

Supplementary Materials are available online.
