# Supplementary figures and images for: Alkaloid Constituents of the Amaryllidaceae Plant Amaryllis belladonna L
Source: Molecules. 2017 Aug 31;22(9):1437. doi: 10.3390/molecules22091437 (PMC6151567; doi:10.3390/molecules22091437)

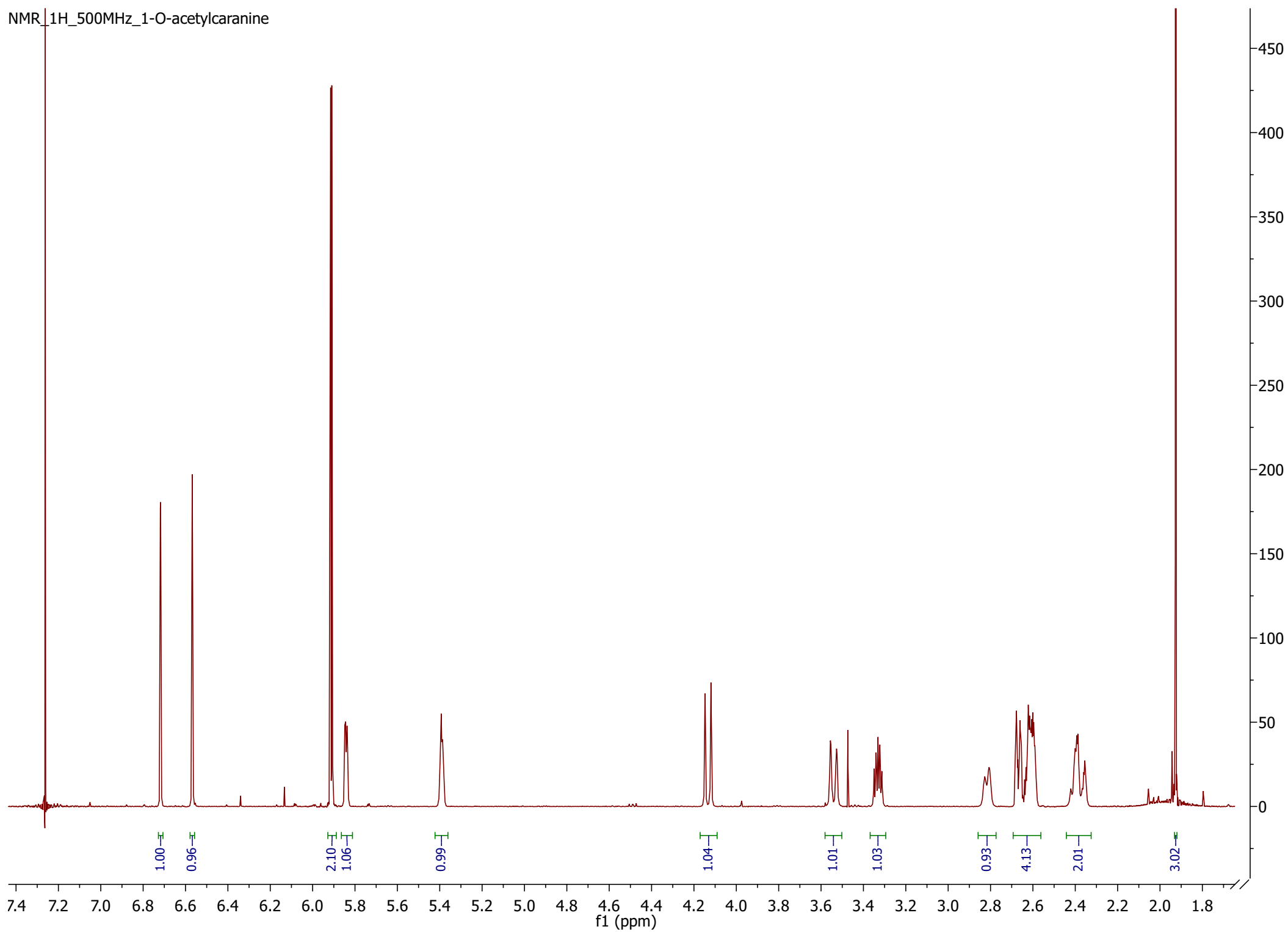

NMR\_COSY\_500MHz\_1-O-acetylcaranine

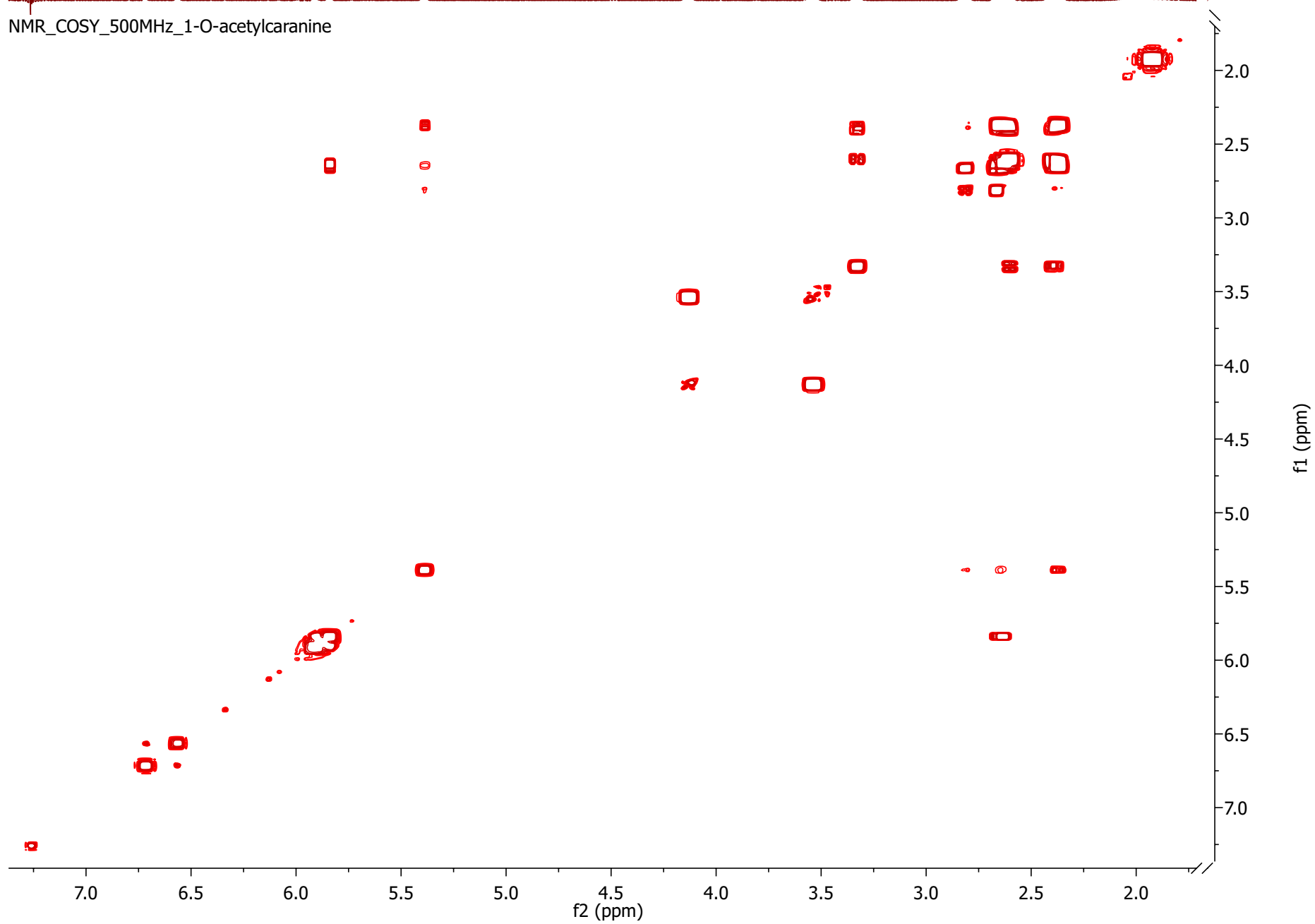

NMR\_NOESY\_500MHz\_1-O-acetylcaranine

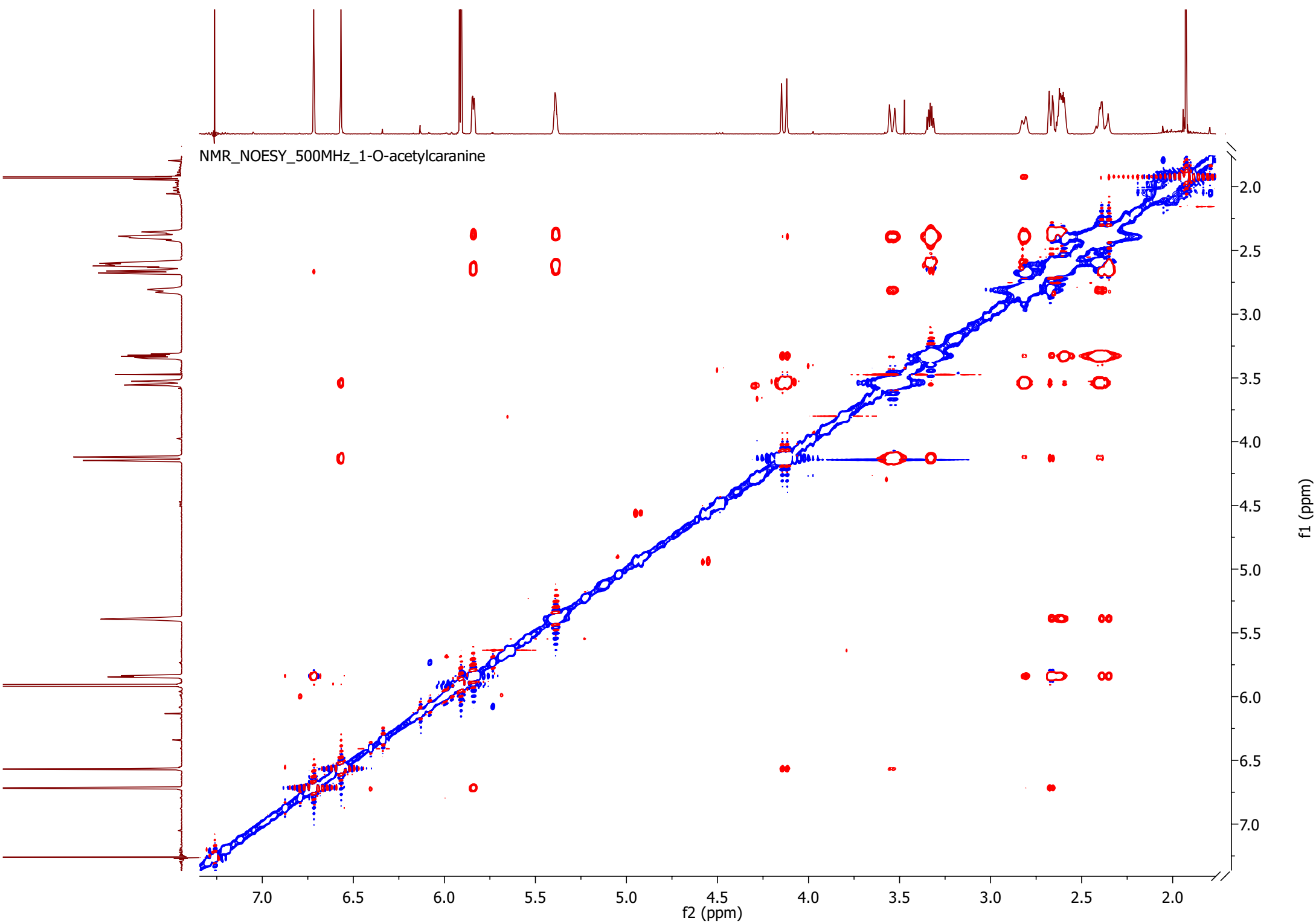

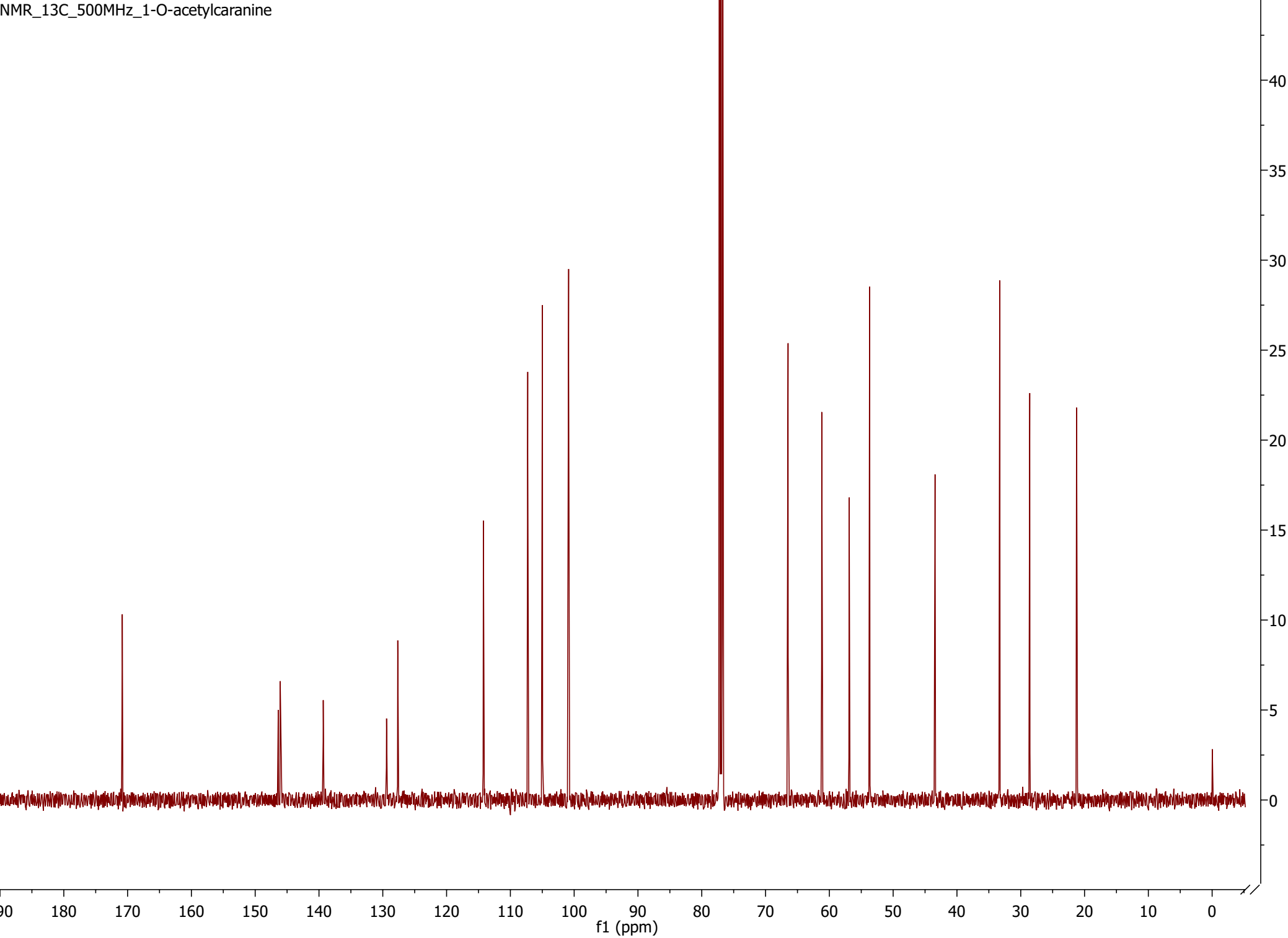

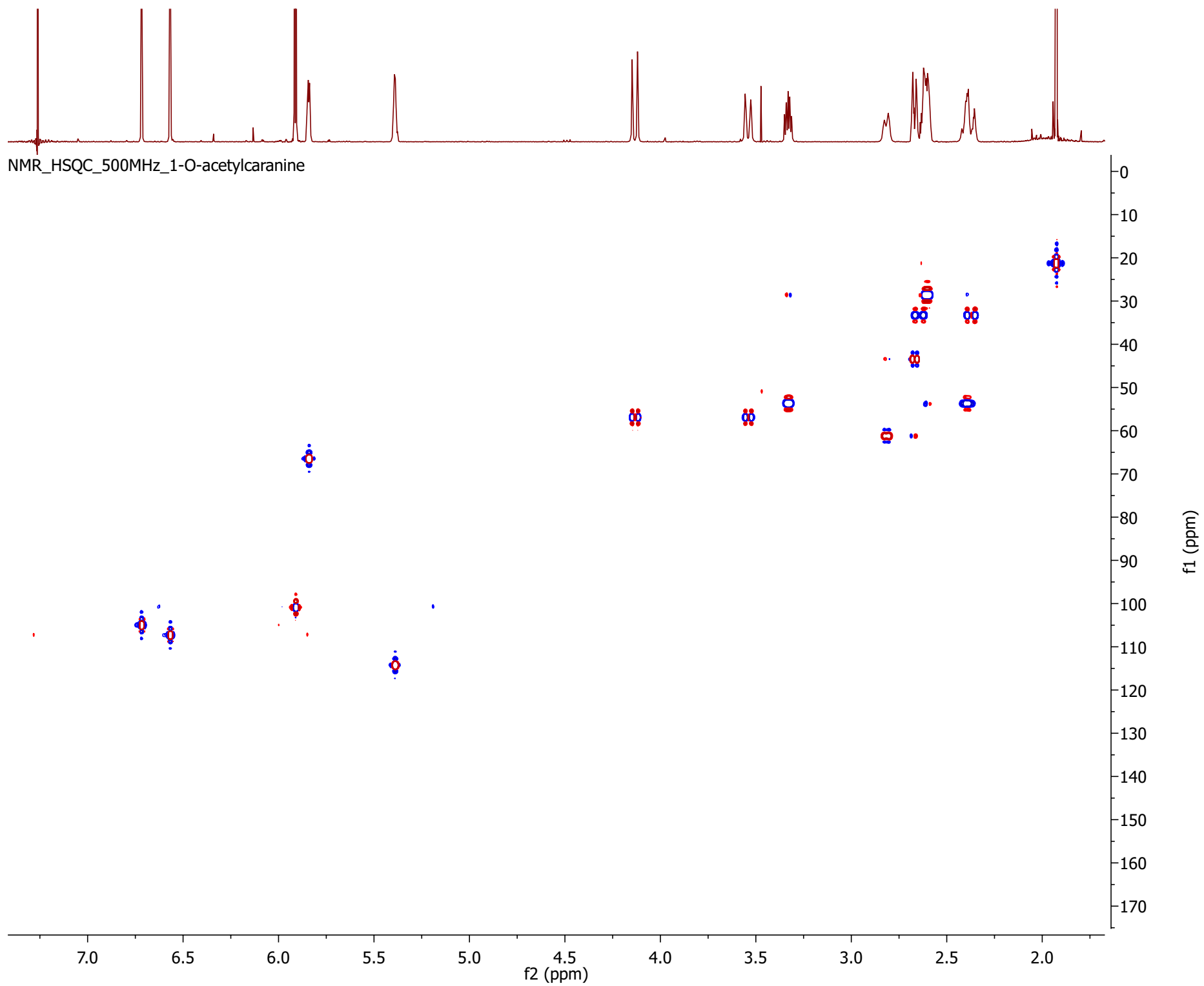

NMR\_HMBC\_500MHz\_1-O-acetylcaranine

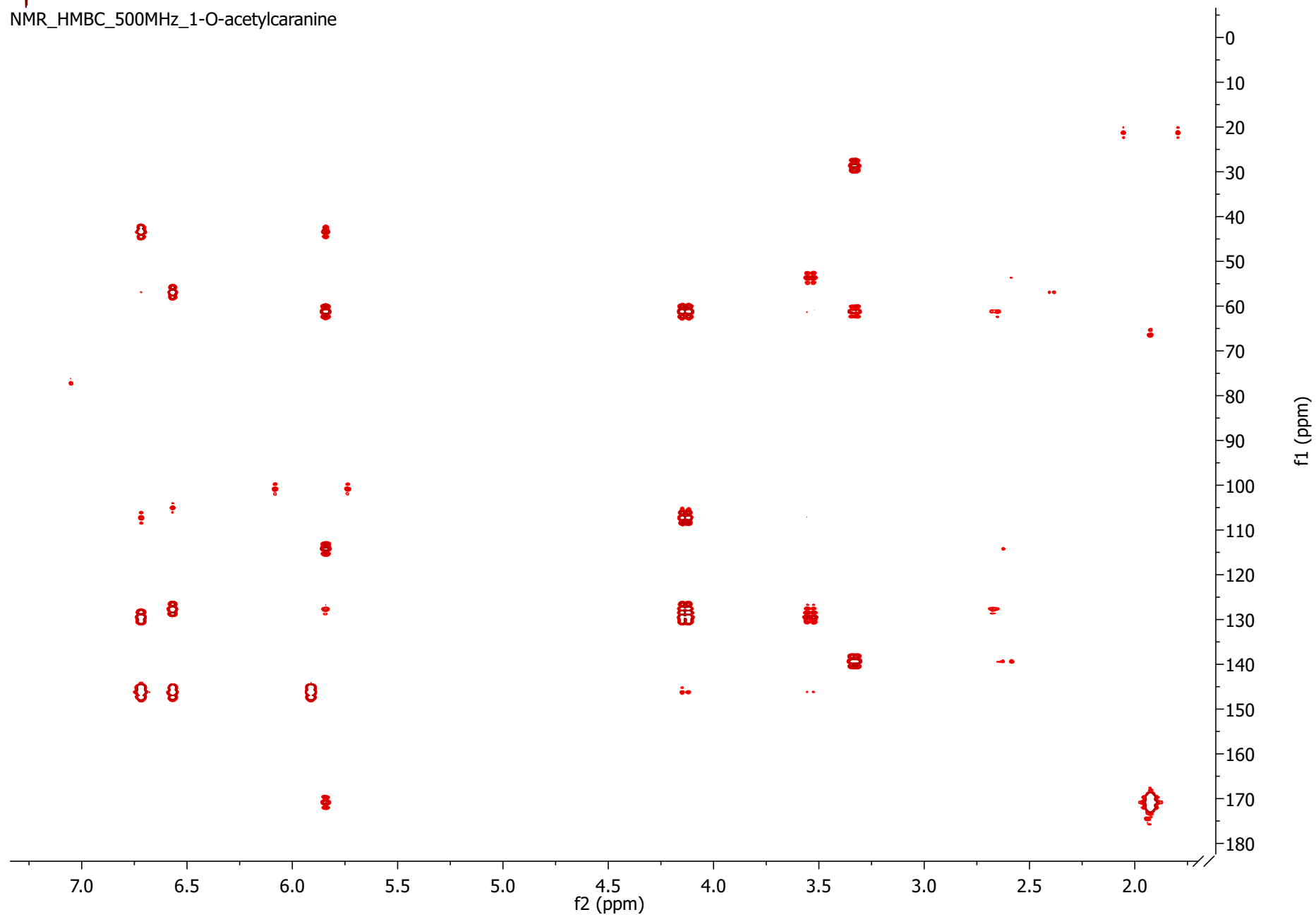

Supplement: Supplementary file 1 [file molecules-22-01437-s001.zip › NMR_1-O-acetylcaranine.pdf]

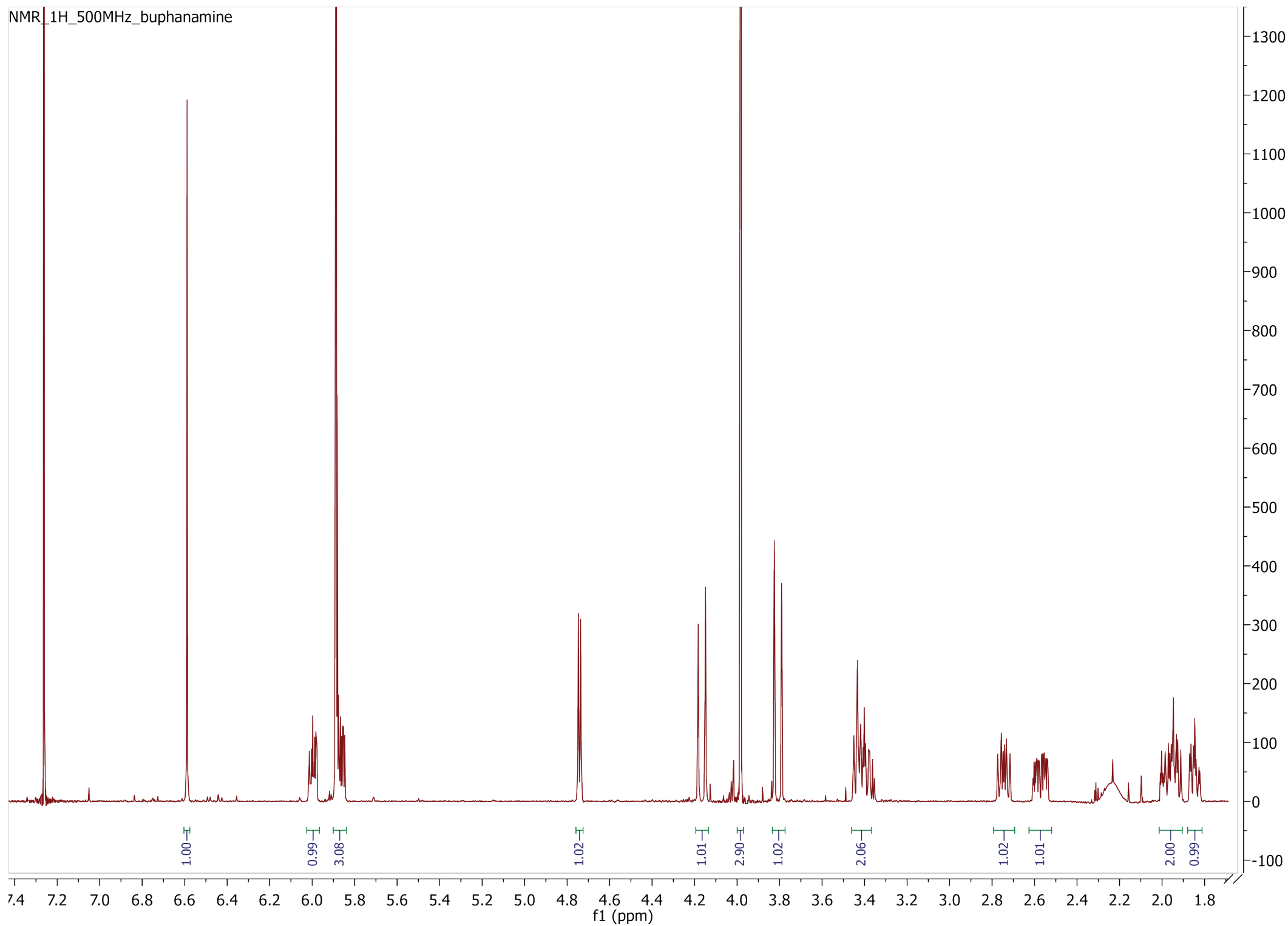

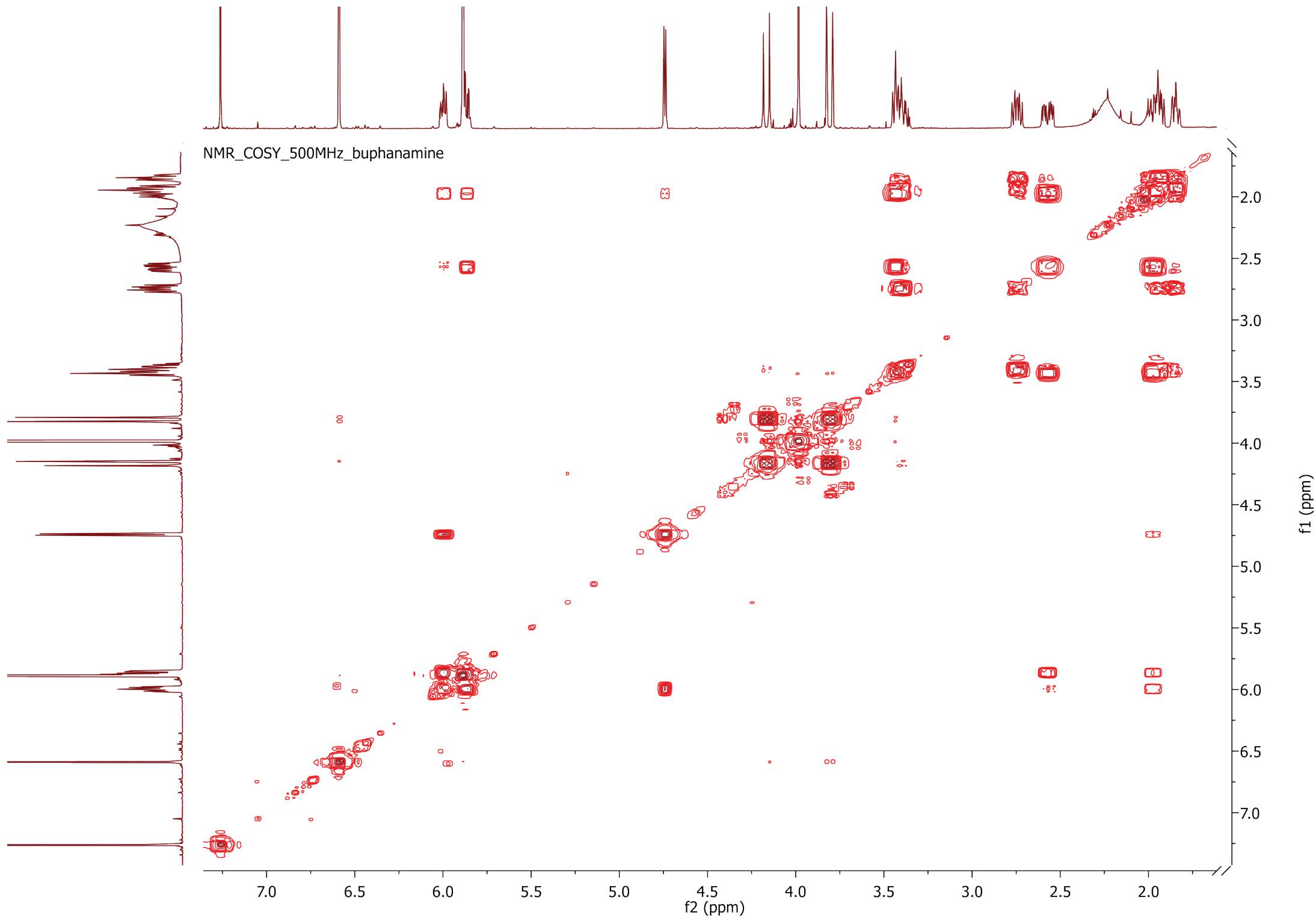

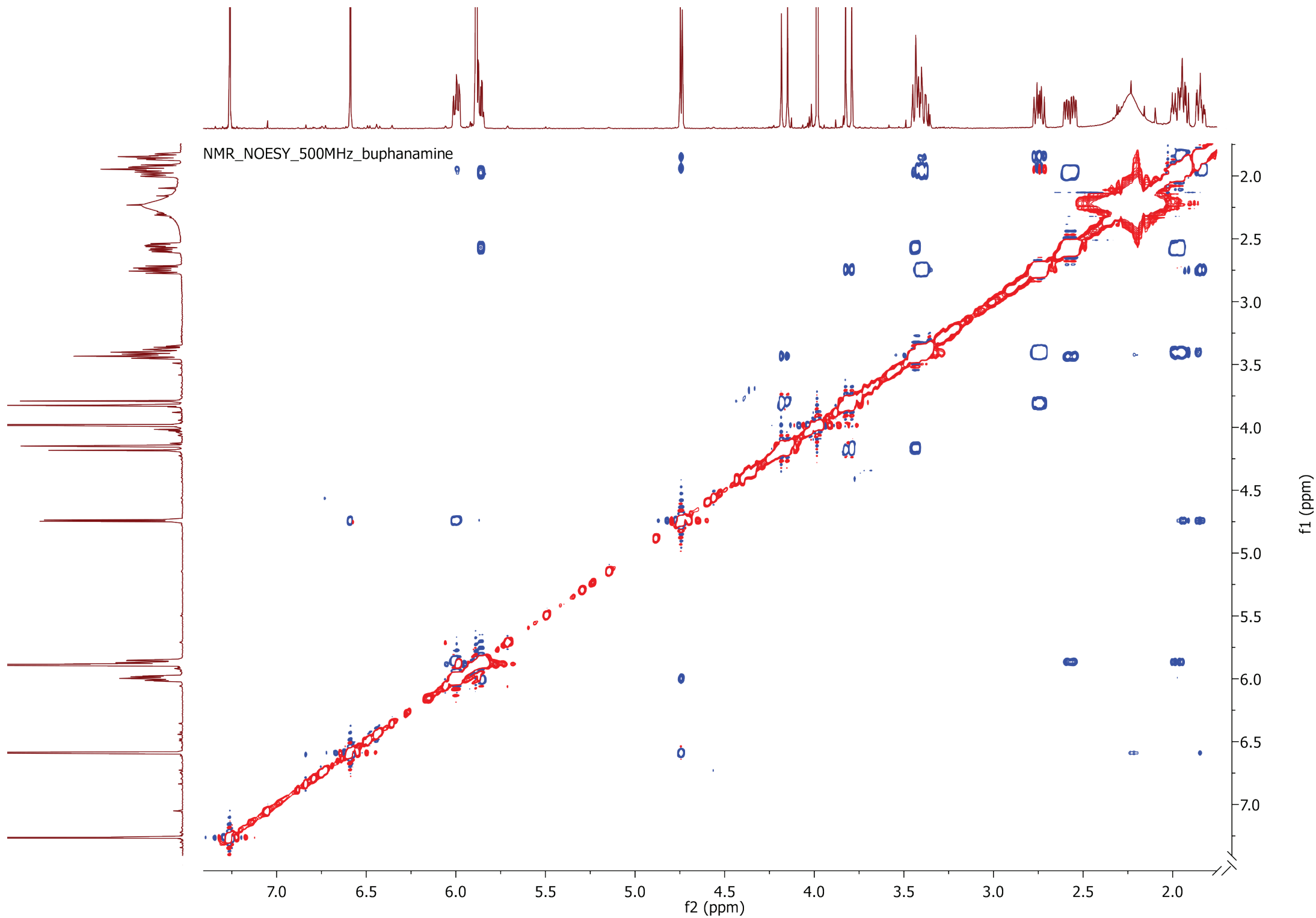

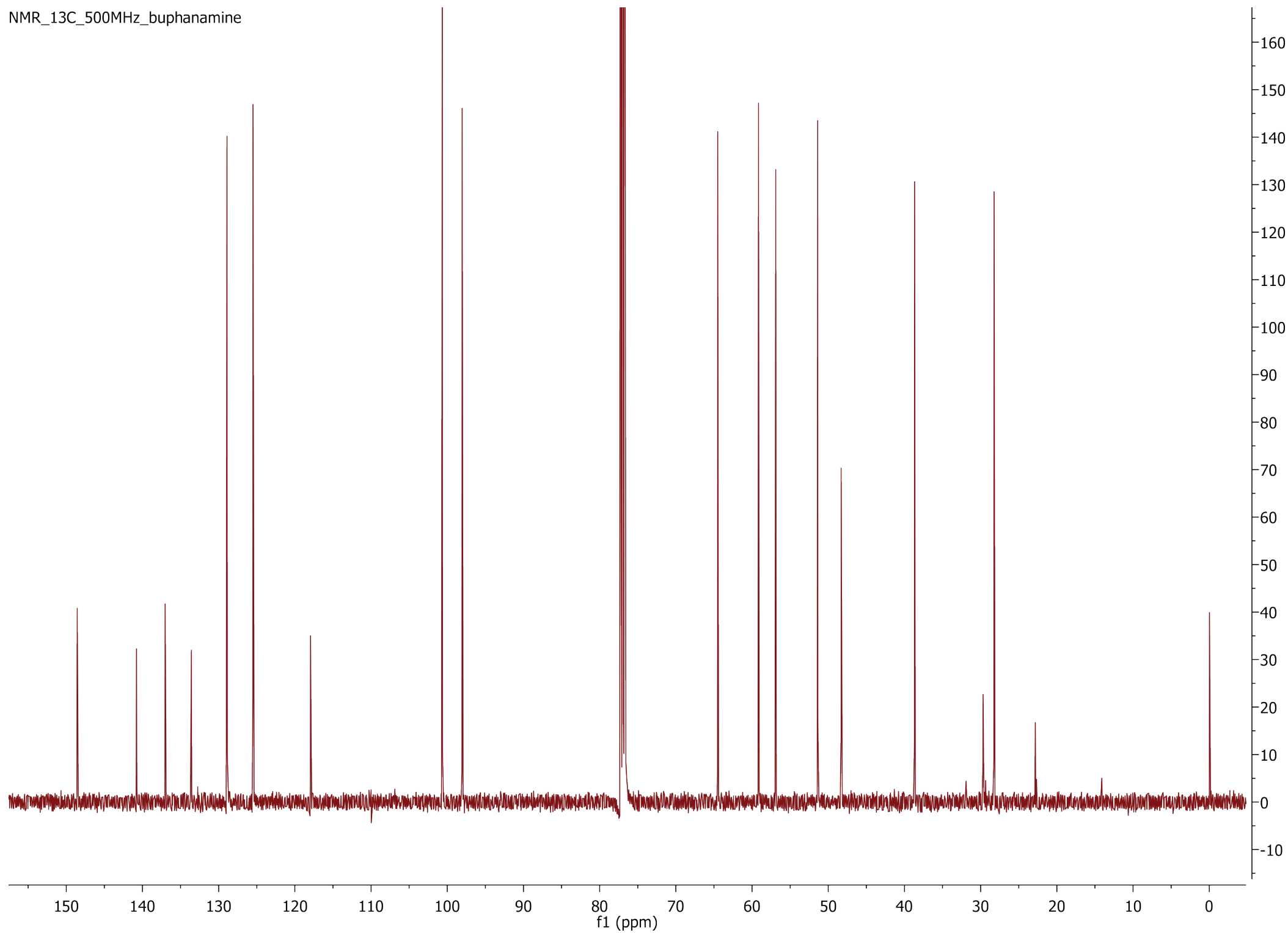

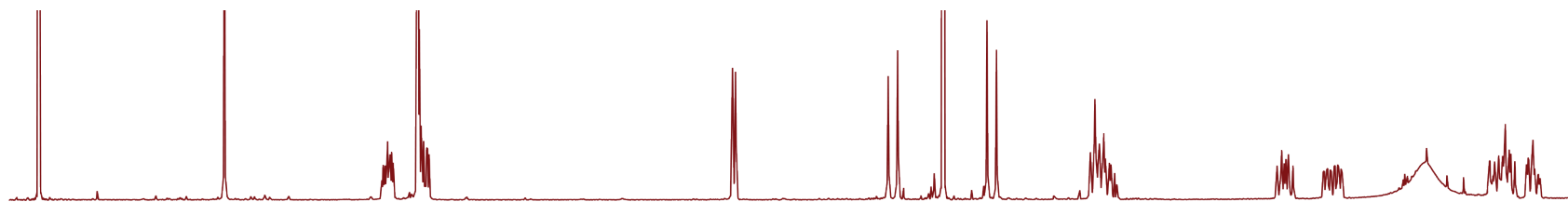

NMR\_HSQC\_500MHz\_buphanamine

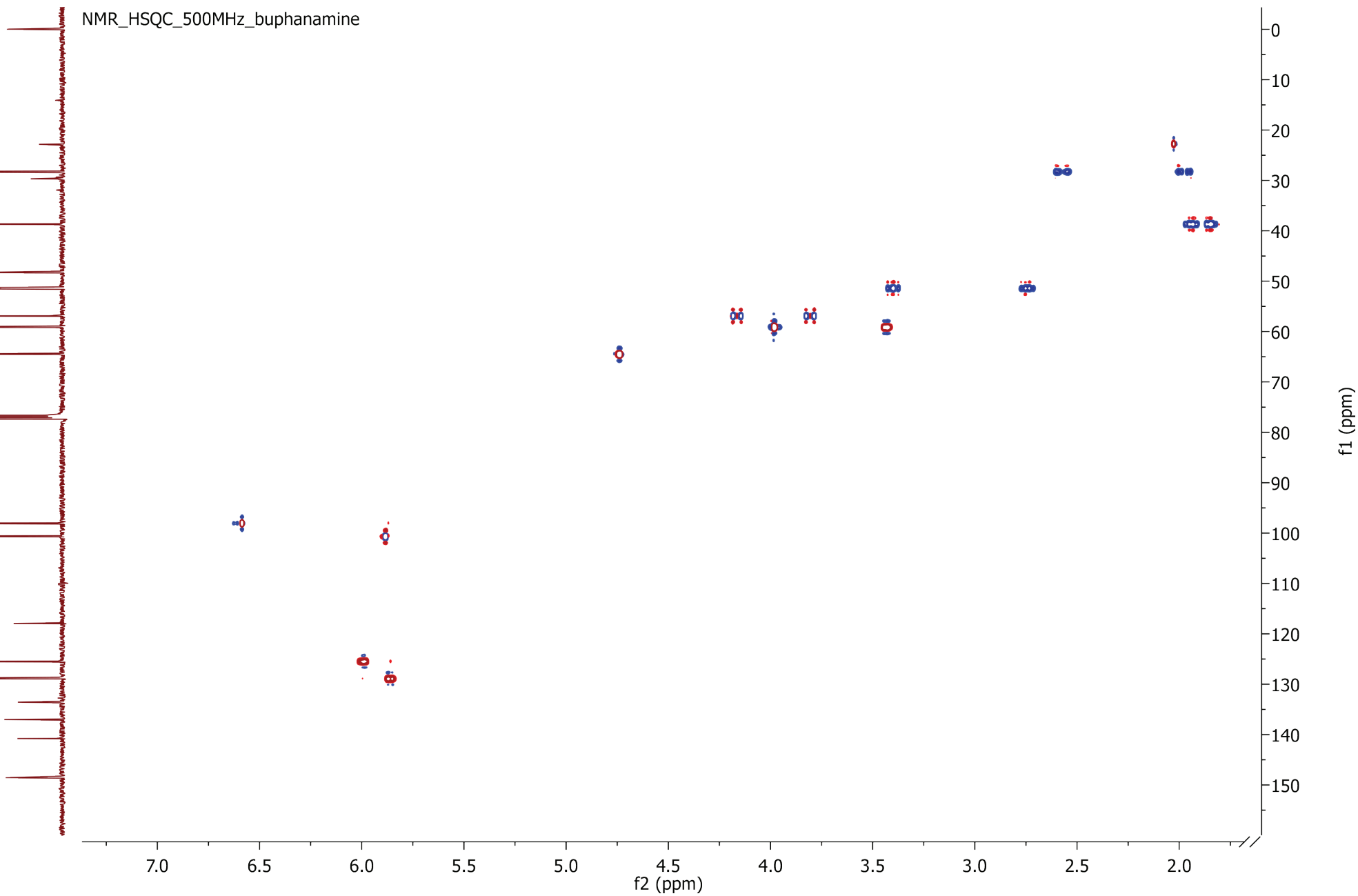

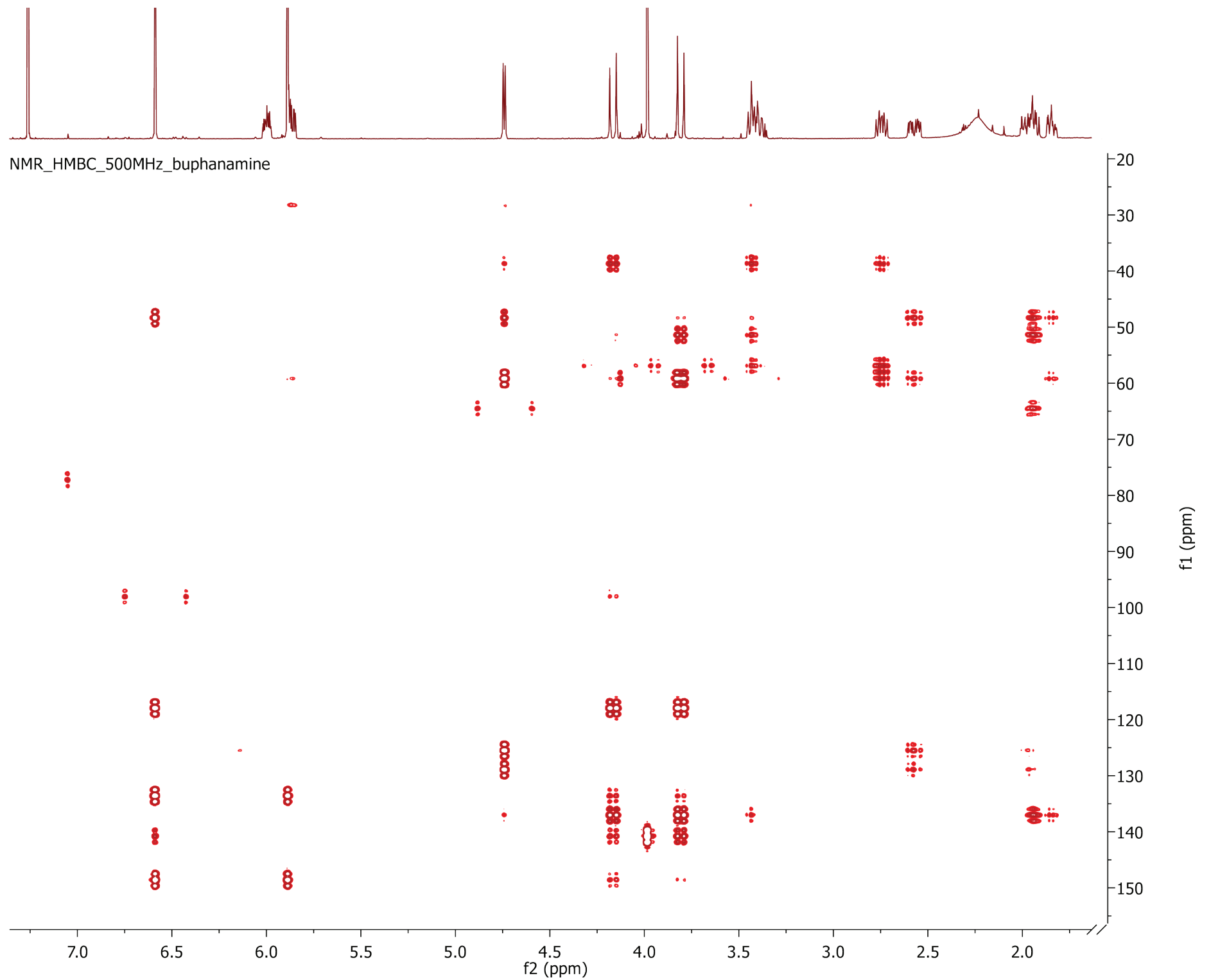

Supplement: Supplementary file 1 [file molecules-22-01437-s001.zip › NMR_buphanamine_.pdf]
